# Development of chronic lung impairment in Mozambican TB patients and associated risks

**DOI:** 10.1186/s12890-020-1167-1

**Published:** 2020-05-07

**Authors:** Celso Khosa, Nilesh Bhatt, Isabel Massango, Khalide Azam, Elmar Saathoff, Abhishek Bakuli, Friedrich Riess, Olena Ivanova, Michael Hoelscher, Andrea Rachow

**Affiliations:** 1 Instituto Nacional de Saúde (INS), Maputo, Mozambique; 2 Center for International Health – CIHLMU, Munich, Germany; 3 Division of Infectious Diseases and Tropical Medicine, University Hospital, LMU Munich, Munich, Germany; 4 German Centre for Infection Research (DZIF), partner site, Munich, Germany

**Keywords:** Tuberculosis (TB), Treatment outcome, Pulmonary function testing, Lung function in TB disease

## Abstract

**Background:**

Pulmonary tuberculosis (PTB) is frequently associated with chronic respiratory impairment despite microbiological cure. There are only a few clinical research studies that describe the course, type and severity as well as associated risk factors for lung impairment (LI) in TB patients.

**Methods:**

A prospective cohort study was conducted at TB Research Clinic of Instituto Nacional de Saúde in Mavalane, Maputo, from June 2014 to June 2016. PTB patients were prospectively enrolled and followed for 52 weeks after TB diagnosis. Lung function was evaluated by spirometry at 8, 26 and 52 weeks after TB treatment initiation, and spirometric values of below the lower limit of normality were considered as LI. Descriptive statistical analysis was performed to summarize the proportion of patients with different lung outcomes at week 52, including type and severity of LI. Risk factors were analysed using multinomial regression analysis.

**Results:**

A total of 69 PTB patients were enrolled, of which 62 had a valid spirometry result at week 52 after TB treatment start. At week 8, 26 and 52, the proportion of patients with LI was 78, 68.9 and 64.5%, respectively, and 35.5% had moderate or severe LI at week 52. The majority of patients with LI suffered from pulmonary restriction. Female sex, low haemoglobin and heavy smoking were significantly associated with LI.

**Conclusion:**

Moderate or severe LI can be observed in a third of cured TB patients. Further research is urgently needed to gain deeper insight into the characteristics of post TB LI, the causal pathways and potential treatment strategies.

## Background

Globally, about seven million new and relapse Tuberculosis (TB) cases were diagnosed in 2018 with a treatment success rate of about 85% [1]. However, in a great proportion of up to 70% of TB patients TB disease does not end with microbiological cure [2–6]. As a consequence of active lung infection with *Mycobacterium tuberculosis (Mtb)*, many patients undergo parenchymal structure damage, including bronchovascular distortion, excessive fibrosis, bronchiectasis and pleural thickening [2]. These anatomical changes can result in chronic chest symptoms and a long-term reduction of overall lung function, including ventilatory and respiratory failure. In the clinical assessment of a relevant proportion of post TB patients, abnormalities in spirometry could be found [7–9]. However, not many studies were performed so far in which the lung function of former TB patients was systematically assessed by spirometry. In the majority of these studies a onetime measurement, usually during or directly after TB treatment, was performed. This approach, however, does only provide limited information on the evolution and chronification of lung impairment (LI) during and after pulmonary TB disease. Further, the scientific evidence on the type and severity of respiratory impairment pattern is scarce.

Ravimohan et al. [2], described several, probably genetically defined, immunological and inflammatory pathways which may result in the destruction of lung tissue and consecutive function impairment. However, the clinical, behavioural or microbiological risk factors for the development of pulmonary sequelae has not been studied in depth yet. In the few existing and relatively small studies partly contradicting evidence was found for an association of post TB lung disease with smoking habits, Human Immunodeficiency Virus (HIV)-status, sex and chest x-ray findings [3, 5, 10–18].

The objective of this study was to prospectively assess the evolution of long-term pulmonary function impairment in spirometry and to get first insights on the associated risk factors in pulmonary TB patients from Maputo, Mozambique. In the absence of a common case definition for chronic lung diseases after TB [19], in this study we used the term “lung impairment (LI)” for spirometric measurements which were below the lower limit of normality (LLN) threshold.

## Methods

### TB study design and population

A prospective cohort study was performed at the Instituto Nacional de Saúde (INS) TB Research Clinic in Maputo, Mozambique, which is located at the premises of the Mavalane Health Center and the National TB Program (NTP) treatment clinic in Mavalane. Mavalane is at the outskirt of Maputo city, an peri-urban area with more than 600.000 inhabitants suffering from a high HIV and TB burden. The study was used as a pilot to collect basic information on the clinical and microbiological characteristics and TB outcomes of TB patients in Maputo. Therefore, no sample size calculation was performed prior to study start.

Pulmonary TB patients were consecutively enrolled from June 14th, 2014 to of May 28th, 2015 and prospectively followed for at least 52 weeks. Study participants were recruited through the NTP treatment clinic in Mavalane, to which they were referred for TB diagnosis and treatment initiation from different health centers in this area. All patients aged 18 years or older with positive Xpert MTB/RIF results and willingness to be tested for HIV were enrolled in this study. Patients, who had been treated for TB in the past 6 months or who had abandoned TB treatment, as well as patients with a Karnofsky score of below 50% [20], or suffering from a medical condition likely to lead to discontinuation of the study, were excluded. TB treatment was administered to all study participants by the Mavalane NTP treatment clinic and according to NTP guidelines. All patients, who tested positive for HIV were referred to comprehensive HIV treatment according to National guidelines.

### Study procedures and data collection

At baseline, data on clinical history, physical examination, laboratory blood assessment and chest x-ray were collected before anti-TB treatment was initiated. Chest x-ray data were analysed as described previously [21]. Follow up visits were performed at weeks 1, 2, 4, 8, 12, 17, 26 and 52 after TB treatment initiation. On all study visits, a clinical examination was performed and sputum was collected for smear microscopy, liquid and solid culture. At 8, 26 and 52 weeks, spirometry assessment was performed using a handheld device (EasyOne ndd®) with individual disposable mouthpieces (Spirette ndd®). American Thoracic Society (ATS)/European Respiratory Society (ERS) guidelines were followed for the measurement of ventilatory parameters and for quality control procedures [22]. Readings for Forced Ventilatory Capacity (FVC) and Forced Expiratory Volume in 1 s (FEV1) were assessed for acceptability (checking the performance of each individual curve for a satisfactory start, middle and end of test) and repeatability (assessment of the variability between the three best curves within one test) according to ATS/ERS guidelines [22] by two investigators, first the investigating physician or nurse and in a second step by the study principal investigators. To ensure data completeness and quality, regular monitoring of study procedures, including spirometry, and of all collected data were performed by external monitors.

### Healthy volunteers

To compare lung function data of study TB patients with that of a local non-TB population, spirometry was also performed in 155 healthy volunteers from Mavalane area between April 20th, and December 6th, 2017. Subjects were either recruited from the same or neighbouring households of the study TB patients, or from the HIV counselling and testing clinic at the Mavalane Health Center. The inclusion and exclusion criteria for the volunteers can be found in S1 table. Collection, monitoring and analysis of spirometry data was performed in the same manner as described for pulmonary TB patients.

### Ethics

All research procedures, including the consenting process, were approved by Comité Nacional de Bioética para Saúde (CNBS), in Mozambique, with the reference 274/CNBS/13 and the Ethics Commission of the Medical Faculty at Ludwig Maximilian University, in Germany. Each study participant and healthy volunteer provided written consent prior enrolment. An impartial witness was included in the consenting process for all illiterate participants. The TB cohort study was registered under ClinicalTrials.gov: NCT02156882.

### Statistical analysis

We analysed the data using STATA version 14 and R 3.5.1. The primary outcome was lung function as measured by spirometry at 52 weeks after TB treatment initiation. For analysis of lung function results, the spirometric prediction equations for South-African populations [23] were applied as reference standard for predicted values for FVC, FEV1, and residual z-scores [22, 23]. In the absence of a South African standard for the predicted value for FEV1/FVC-ratio we used the prediction equation published by the Global Lung Initiative (GLI) [24, 25]. In line with most recent guidelines [24, 26], we defined the 5th percentile (this is 1.64 standard deviations below the predicted value and is represented by a residual z-score of below − 1.64) as the LLN for lung function. That means, that study participants with spirometric values for FVC and/or FEV/FVC-ratio which were below the 5th percentile of the predicted value were considered to have abnormal lung function, thus were classified as having LI. Concretely, a FVC-value below the 5th percentile of predicted constituted the diagnosis of restrictive lung impairment and participants with a value for the FEV1/FVC-ratio of below the LLN were diagnosed with obstructive lung impairment, respectively. In participants with mixed lung impairment both values for FVC and FEV1/FVC-ratio were below the LLN. Severity grading was done as follows; mild: FVC or FEV1/FVC > 85% LLN, moderate: FVC or FEV1/FVC 55–85% of LLN, severe: FVC or FEV1/FVC < 55% of LLN [27]. Comparison of proportions using Chi-Square Test and medians (means) using Wilcoxon Rank Sum test (t tests (paired/unpaired)) as well as Kruskal Wallis test with post hoc testing for pairwise comparison was performed [28]. Lung function z-scores for different participant sub-groups were visualized using kernel density plots. Multinomial regression was applied to estimate the relative risk ratios (RRRs) for the individual risk factors in the outcome categories mild impairment or moderate/severe impairment, considering the unimpaired group as reference [29].

## Results

### Description of study population

**Fig. 1:**
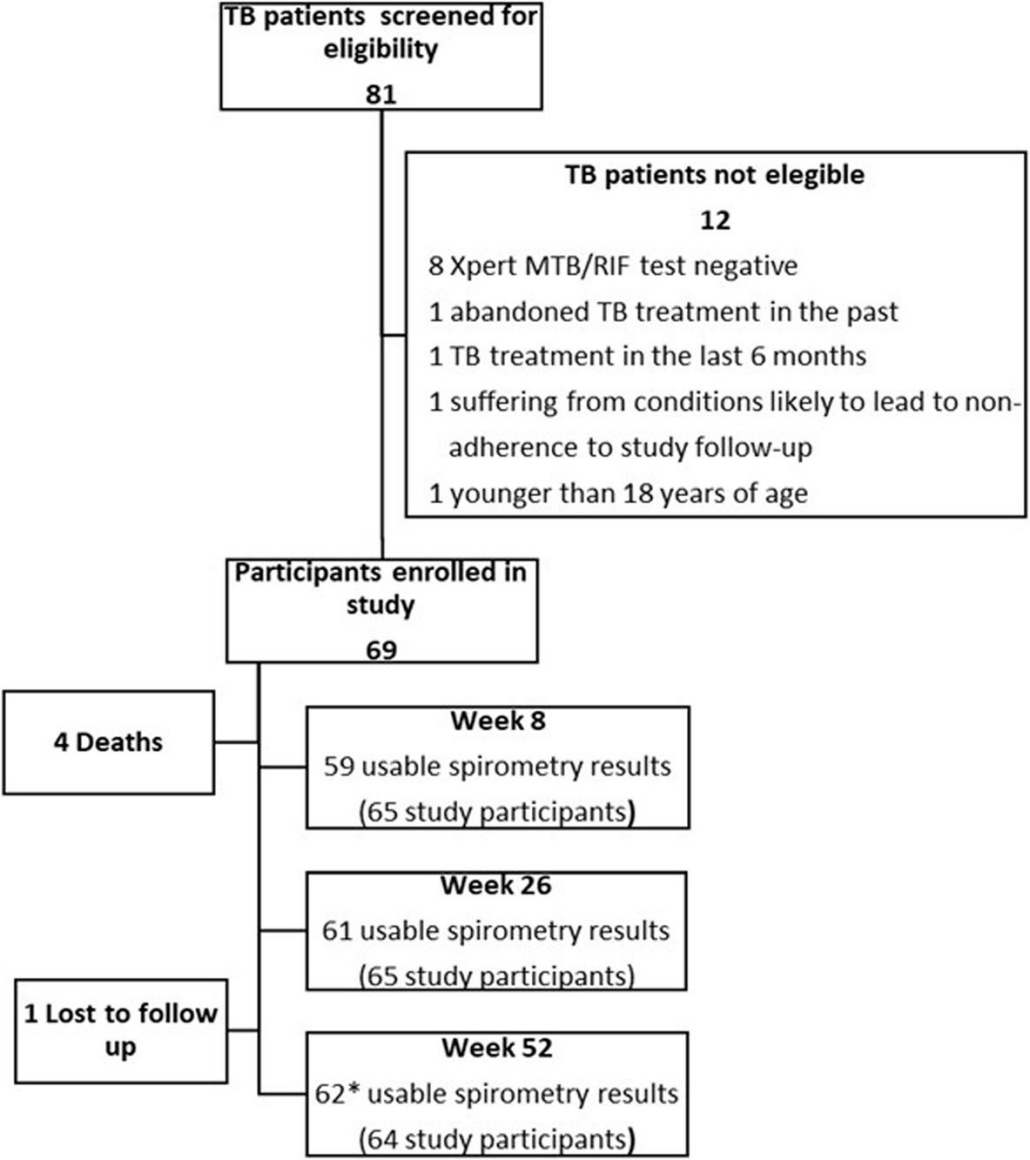
Study flow diagram. * Participants included in the final analyses

In total, 81 TB patients were screened for the study. Twelve subjects who did not meet all eligibility criteria were excluded. Of the enrolled 69 participants, 62 participants with spirometry results at week 52 were included in the final analysis (Fig. 1). Males were the majority of study subjects with 67.7% (42/62). The median age was 29.5 years (Interquartile range (IQR): 25–40), and 74.2% (46/62) of participants were below 40 years old. HIV-coinfection was found in 62.9% (39/62), with a CD4 count of below 200 helper cells/μl in almost half (46.2%, 18/39) of HIV-positive patients. At baseline, the majority of participants had an abnormally low haemoglobin level 83.9% (52/62) and an increased level (above 50 mg/dl) of C-reactive protein (CRP) 69% (40/58), the median Body Mass Index (BMI) was 19.2 kg/m^2^. Thirty-seven percent (23/62) of study participants have ever smoked, all of these were male, and about half of the study population (53.3%, 32/69) reported to regularly consume critical amounts of alcohol (Table 1).

**Table 1:** Baseline characteristics of TB cohort participants, included in final analysis

Characteristic	TB patients in final analysis (***N*** = 62)
**Sex**	
Male, % (n/N)	67.74 (42/62)
**Age**	
Median (IQR)	29.5 (25, 40)
< 40 years old, % (n/N)	74.19 (46/62)
**HIV-status**	
Positive, % (n/N)	62.90 (39/62)
CD4- cells < 200/μl, % (n/N)	46.15 (18/39)
CD4- cells < 300–499/μl, % (n/N)	38.46 (15/39)
CD4- cells ≥500/μl, % (n/N)	15.38 (6/39)
**History of TB**	
Yes, % (n/N)	6.45 (4/62)
**RIF resistance**	
Present, % (n/N)	6.45 (4/62)
**Chronic respiratory diseases**^**a**^	
Present, % (n/N)	4.84 (3/62)
**Haemoglobin**	
Median male, g/dl (IQR)	11.70 (10.2, 12.6)
Median female, g/dl (IQR)	9.90 (9.45, 11.00)
**Anaemia**^**b**^	
No, % (n/N)	16.13 (10/62)
Mild, % (n/N)	37.10 (23/62)
Moderate, % (n/N)	41.94 (26/62)
Severe, % (n/N)	4.84 (3/62)
**C-reactive protein (CRP)**^**b**^**,**^**c**^	
Median, mg/dl (IQR)	76.78 (44.0, 106.95)
< 50 mg/dl, % (n/N)	31.03 (18/58)
51–100 mg/dl, % (n/N)	39.66 (23/58)
> 100 mg/dl, % (n/N)	29.31 (17/58)
**BMI**^**b**^	
Median (IQR)	19.23 (17.51, 20.50)
< 18.5, % (n/N)	41.94 (26/62)
**Culture conversion**	
until week 8	56.45 (35/62)
until week 26	93.55 (58/62)
**Overall affected lung**^**c**^	
%, median (IQR)	15 (15, 20)
**Smoking**	
Ever smoked, % (n/N)	35.48 (22/62)
**Pack Years**^**b**, **c**^	
< 10	59.09 (13/22)
≥ 10	31.82 (7/22)
**Alcohol consumption**^**b**^	
Ever alcohol, % (n/N)	77.42 (48/62)
Amount of alcohol, median, g/week (IQR)	80.0 (40, 240)
Critical alcohol consumption^**c**^, % (n/N)	53.33 (32/60)

^**a**^Chronic respiratory diseases (asthma or COPD),

^**b**^missing observations: CRP for 4 participants, Pack Years (PY) for 2 participants, amount of alcohol consumption for 2 participants

^**c**^definitions:

- Anaemia, definition according to WHO, non-anaemia: 12 mg/dl or higher (women) or 13 mg/dl or higher (men), mild: 11.0–11.9 g/dl (women) and 11.0–12.9 g/dl (men), moderate: 8.0 g/dl-10.9 g/dl (both sexes), severe: < 8.0 g/dl (both sexes) [30]

- CRP: normal range: 0.0 md/dl − 10 mg/dl, only one participant had a CRP value below 10 mg/dl at baseline

- X-ray scoring system for reporting overall affected lung, according to Ralph et al. [21]

- Pack Years (PY): lifetime exposure to cigarette smoking, e.g. ≥10PY means that the respective study participant smoked an equivalent of one pack of cigarettes every day for at least 10 years (e.g. 1 pack of cigarettes on every day for 10 years, or 2 packs of cigarettes on every day for 5 years, or ½ packs of cigarettes on every day for 20 years) at baseline study visit. PY were only calculated for those 20 male study participants who reported to have “ever smoked” during their lifetime and provided information on PY

- Critical alcohol consumption: in men (women) more than 60 g (30 g) alcohol per occasion of alcohol drinking and/or more than 150 g (80 g) alcohol per week, according to the International Alliance for Responsible Drinking for general population [31]

### Spirometry results

In Table 2, the spirometry results (FVC, FEV1 and FEV1/FVC-ratio) on the different study visits are summarized. While at week 8 visit 78% of all study participants had LI, the proportion declined to 64.52% at week 52. In line with that, the mean values for FVC and FEV1 (in litre, as percentage of predicted and z-scores) were significantly increasing for the whole study population from week 8 until week 52. Among the participants with LI at week 52, only 67.5% (2.66 l, z-score = − 2.64) and 66.3% (2.09 l, z-score = − 2.47) of the predicted FVC and FEV1, respectively, were achieved on average. The z-scores and means of the FEV1/FVC ratio were above − 1.64 and 0.70, respectively, in all sub-groups and at all study visits.

**Table 2:** Spirometry results of study participants at week 8, 26 and 52 after TB treatment initiation, included in final analysis (*N* = 62)

	Week 8^**a**^			Week 26			Week 52		
	All (***N*** = 59)	LI (***N*** = 46)	No LI (***N*** = 13)	All (***N*** = 61)	LI (***N*** = 42)	No LI (***N*** = 19)	All (N = 62)	LI (***N*** = 40)	No LI (***N*** = 22)
**% of N**	100.00	77.97	22.03	100.00	68.85	31.15	100.00	64.52	35.48
**FVC in l (SD)**	2.79 (0.76)	2.56 (0.60)	3.61 (0.70)	2.91 (0.79)	2.57 (0.60)	3.66 (0.62)	3.05 (0.78)	2.66 (0.58)	3.77 (0.55)
**FVC in % of pred. (SD)**	69.00 (15.18)	63.68 (11.84)	87.85 (9.75)	72.00 (15.46)	64.27 (11.05)	89.09 (8.41)	75.46 (14.96)	67.48 (11.21)	89.99 (8.62)
**FVC z-score (SD)**^**a**^	−2.52 (1.30)	−2.96 (1.06)	−0.98 (0.78)	−2.25 (1.24)	− 2.88 (0.88)	− 0.87 (0.66)	−1.99 (1.21)	−2.64 (0.90)	− 0.80 (0.68)
**FEV1 in l (SD)**	2.27 (0.68)	2.06 (0.54)	3.01 (0.64)	2.33 (0.70)	2.02 (0.52)	3.01 (0.54)	2.44 (0.70)	2.09 (0.52)	3.07 (0.52)
**FEV1 in % of pred. (SD)**	69.54 (16.64)	63.54 (12.81)	90.76 (9.86)	71.39 (17.72)	62.60 (13.06)	90.82 (8.96)	74.82 (16.63)	66.28 (12.89)	90.35 (10.15)
**FEV1 z-score (SD)**^**a**^	−2.26 (1.28)	−2.71 (1.02)	−0.68 (0.76)	−2.10 (1.27)	− 2.74 (0.90)	− 0.68 (0.67)	−1.85 (1.21)	−2.47 (0.93)	−0.72 (0.74)
**Ratio (FEV1/FVC)**	81.14 (9.74)	80.66 (10.69)	82.85 (5.11)	79.31 (8.69)	78.01 (9.55)	82.17 (5.64)	79.44 (7.95)	78.50 (9.28)	81.16 (4.31)
**FEV1/FVC z-score (SD)**^**a**^	−0.42 (1.59)	−0.49 (1.75)	− 0.17 (0.74)	−0.77 (1.22)	− 0.98 (1.33)	−0.31 (0.79)	− 0.75 (1.28)	−0.92 (1.50)	− 0.46 (0.69)

^**a**^valid spirometry data available only from 59 participants at week 8 and 61 participants at week 26

*SD* standard deviation, *pred* predicted, *LI* lung impairment, *%* percentage, *N* number of subjects in (sub-) group

There is a statistical evidence for a difference between the mean FVC at week 8 and week 52, *p* = 0.046, and no statistical difference between the mean FVC at week 8 and week 26, *p* = 0.36, and the mean FVC at week 26 and week 52, *p* = 0.23. There is no statistical difference for mean FEV1 between different study visits: week 8 versus week 26, *p* = 0.46, week 26 versus week 52, *p* = 0.34, and week 8 versus week 52, p = 0.12

**Fig. 2:**
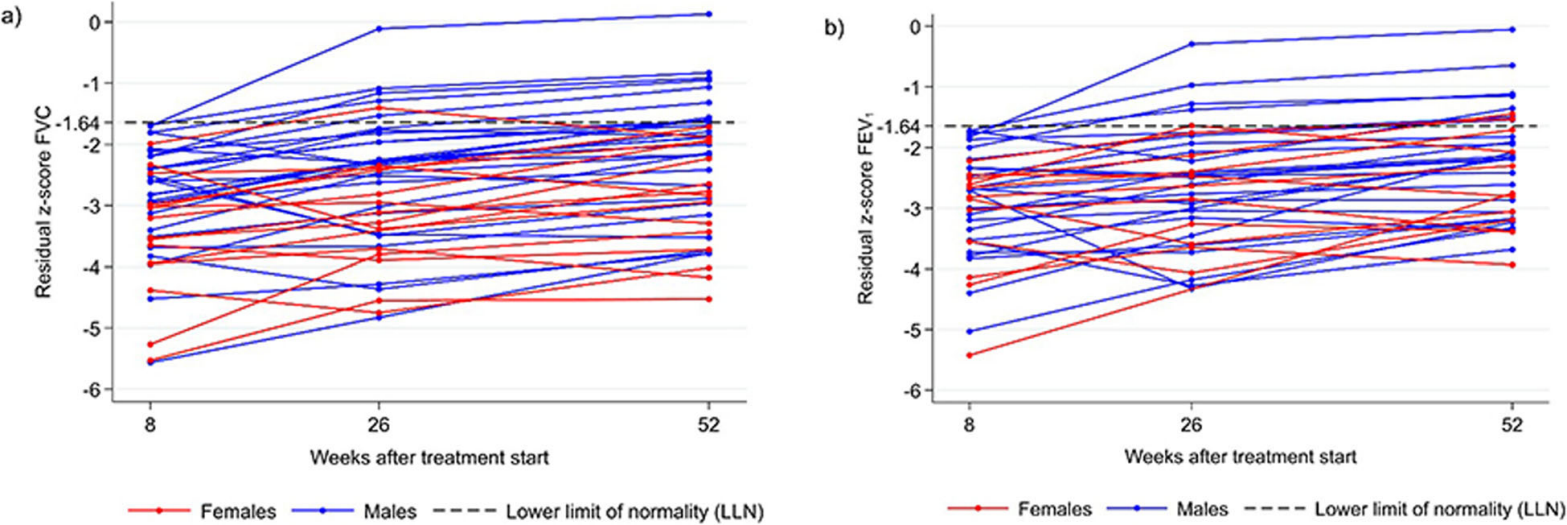
Z-scores for FVC (**a**) and FEV1 (**b**) at week 8, 26 and 52 of those participants with LI at week 8. Change in residual z-scores for FVC (2a) and FEV1 (2b) over time in study participants with abnormal values for FVC and/or FEV1 (residual z-score < − 1.64) at week 8. Residual z-scores (adjusted for sex, height and age) were calculated based on South African reference standard [23]

Figure 2 shows the development of respiratory parameters over time for those participants, who had abnormal z-scores for FVC or FEV1 at week 8. While for both lung function parameters the z-scores were improving in the majority of these participants, only in few of them (8/62, 12.9%) this led to a normalization of both parameters until week 52. Opposed to men, almost all women with abnormal FVC or FEV1 at week 8 had still impaired lung function at week 52. In those study participants with normal spirometry values at week 8, three and four subjects developed abnormal values for FVC or FEV1, respectively, until week 52 (S1 fig).

### Type and severity of LI

**Fig. 3:**
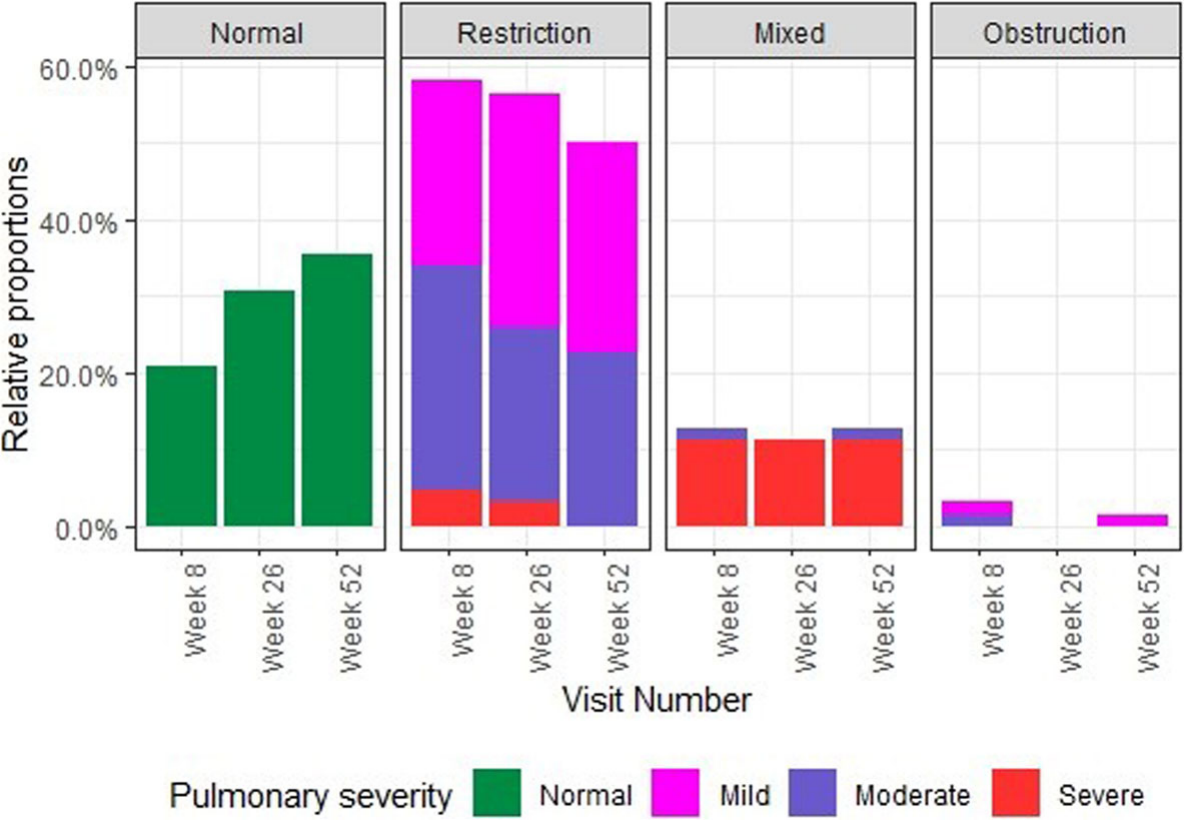
Proportions of types and severity grades of LI at different study visits. Week 8: *N* = 62, normal: 13 (20.97%), mild restriction: 15 (24.19%), mild obstruction: 1 (1.61%), moderate restriction: 18 (29.03%), moderate obstruction: 1 (1.61%), moderate mixed: 1 (1.61%), severe restriction: 3 (4.84%), severe mixed: 7 (11.29%), missing data: 3 (4.84%). Week 26: N = 62, normal: 19 (30.65%), mild restriction: 19 (30.65%), moderate restriction: 14 (22.58%), severe restriction: 2 (3.23%), severe mixed: 7 (11.29%), missing data: 1 (1.61%). Week 52: N = 62, normal: 22 (35.48%), mild restriction: 17 (27.42%), mild obstruction: 1 (1.61%), moderate restriction: 14 (22.58%), moderate mixed: 1 (1.61%), severe mixed: 7 (11.29%)

The majority of subjects with LI (77.5%, 31/40) at week 52 suffered from pulmonary restriction (Fig. 3). In addition, eight (20.0%) participants had mixed LI and one (4.0%) had obstruction at week 52. Not only the overall proportion of study participants with any LI declined from week 8 (79.0%) to week 52 (64.5%), but also the proportion of participants with moderate or severe pulmonary restriction went down from 48.4% (week 8) to 35.5% (week 52).

### Risk factors analysis

We analysed several risk factors for their association with the development of LI at week 52 (Table 3, for numbers and proportions in different sub-groups refer to S2 table). Women had a four to five times higher risk for developing moderate/severe (*p* = 0.05) or mild (*p* = 0.04) LI compared to men. A positive HIV-status was not significantly associated with moderate/severe LI, although there seemed to be an effect on the development of mild LI. Interestingly, among the HIV-positives, those with more than 200 helper cels/μl were more likely to develop mild (RRR: 3.60, *p* = 0.12) or moderate/severe (RRR: 1.87, *p* = 0.43) LI, but this effect was not statistically significant. Further, each unit increase in baseline haemoglobin of one g/dl reduced the relative risk of LI by 33% (mild, *p* = 0.02) and 31% (moderate/severe, *p* = 0.03), respectively. In men, “Ever smoking” was associated with a reduced risk of developing any type of LI (mild LI, RRR: 0.15, *p* = 0.04; moderate/severe LI, RRR: 0.71, *p* = 0.93). However, in the analysis of amount of cigarettes smoked in life (PY), those who smoked at least one pack of cigarettes daily for ten or more years (≥10PY) had a higher risk for developing severe post TB LI (RRR: 7.5, *p* = 0.06) compared to those who smoked less (< 10PY). Also, a low BMI, a delayed culture conversion and critical alcohol consumption increased the risk for abnormal lung function at week 52. These associations, however, were not statistically significant.

**Table 3:** Risk factors for LI at week 52

Baseline^**a**^	Comparator^**a**^	Severity grade of LI	Relative Risk Ratio (RRR)	95% CI for RRR	***p***-value
Male	Female	mild	5.07	1.10, 23.45	0.04
		moderate/severe	4.38	0.99, 19.36	0.05
Age < 40 years	Age ≥ 40 years	mild	0.53	0.11, 2.52	0.43
		moderate/severe	1.24	0.34, 4.56	0.74
BMI ≥18.5	BMI < 18.5	mild	2.19	0.61, 7.81	0.23
		moderate/severe	1.00	0.29, 3.42	0.99
HIV-negative	HIV-positive	mild	1.79	0.47, 6.85	0.39
		moderate/severe	1.00	0.30, 3.33	0.99
CD4 < 200/μl	CD4 ≥ 200/μl	mild	3.60	0.71, 18.25	0.12
		moderate/severe	1.87	0.39, 8.89	0.43
Culture conversion until week 8	No culture conversion until week 8	mild	1.71	0.47, 6.24	0.41
		moderate/severe	2.57	0.75, 8.78	0.13
Culture conversion until week 26	No culture conversion until week 26	mild	2.63	0.22, 31.57	0.45
		moderate/severe	1.00	0.06, 17.07	0.99
Never smoked (males only)	Ever smoked (males only)	mild	0.15	0.02, 0.89	0.04
		moderate/severe	0.71	0.19, 3.58	0.93
< 10 Pack Years^b^	≥10 Pack Years^b^	mild	–	–	–
		moderate/severe	7.50	0.92, 61.05	0.06
No critical alcohol consumption^b^	Critical alcohol consumption^b^	mild	0.69	0.14, 3.40	0.65
		moderate/severe	1.50	0.34, 6.59	0.59
C-reactive protein < 100 mg/dL	C-reactive protein ≥100 mg/dL	mild	0.77	0.19, 3.11	0.72
		moderate/severe	0.58	0.15, 2.26	0.43
Haemoglobin, per 1 g/dl increase at baseline		mild	0.67	0.47, 0.95	0.02
		moderate/severe	0.69	0.49, 0.96	0.03
Overall affected lung, each 1% increase		mild	0.97	0.90, 1.05	0.43
		moderate/severe	1.01	0.95, 1.08	0.68

Risk factor analysis using univariable multinomial regression model, comparing subjects with no impairment at week 52 (*n* = 24) with those who have mild impairment (n = 22) or moderate/severe impairment (*n* = 16) at week 52. The absolute numbers and proportions of subjects in each individual stratum (baseline, comparator, mild LI and moderate/severe LI) can be found in S2 table

^**a**^missing observations: CRP for 4 participants, Pack Years (PY) for 2 participants, amount of alcohol consumption for 2 participants

^b^definitions:

- Pack Years (PY): lifetime exposure to cigarette smoking, e.g. ≥10PY means that the respective study participant smoked an equivalent of one pack of cigarettes every day for at least 10 years (e.g. 1 pack of cigarettes on every day for 10 years, or 2 packs of cigarettes on every day for 5 years, or ½ packs of cigarettes on every day for 20 years) at baseline study visit. PY were only calculated for those 20 male study participants who reported to have “ever smoked” during their lifetime and provided information on PY

- Critical alcohol consumption: in men (women) more than 60 g (30 g) alcohol per occasion of alcohol drinking and/or more than 150 g (80 g) alcohol per week, according to the International Alliance for Responsible Drinking for general population [31]

### Comparison with healthy volunteers

**Fig. 4:**
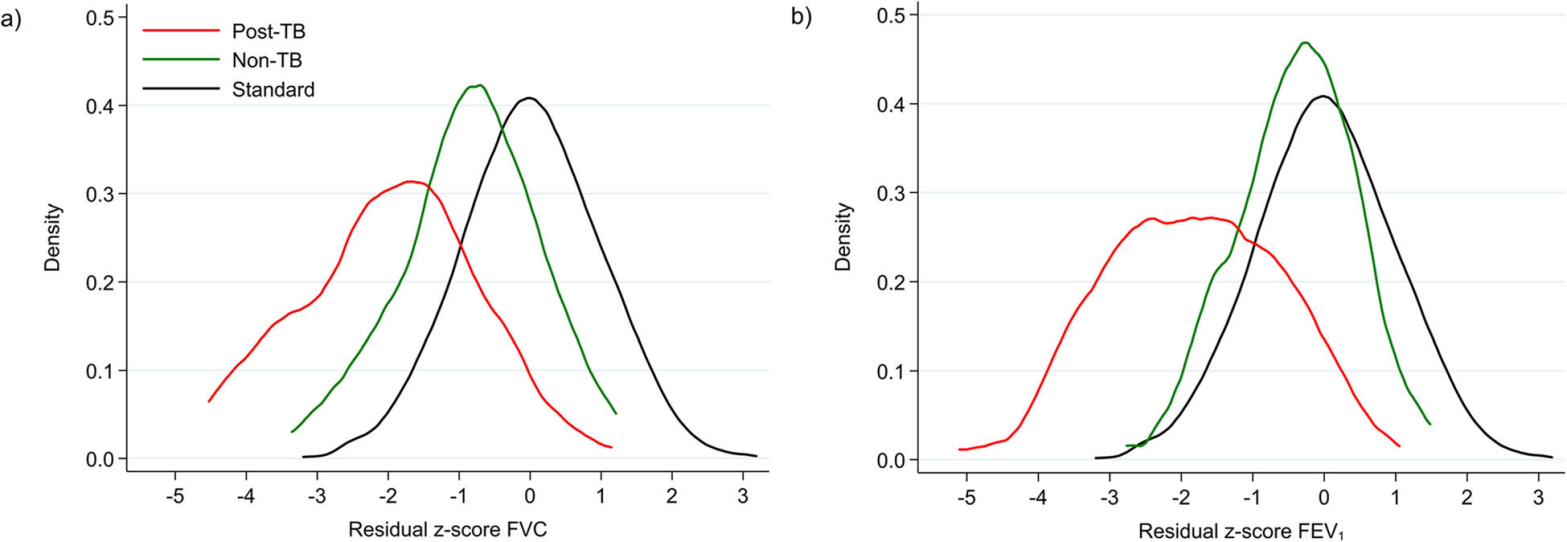
Density plots of z-scores for FVC (**a**) and FEV1 (**b**) by study populations. Density (frequency) distribution of residual z-scores for FVC (**a**) and FEV1 (**b**) by different study populations (TB cohort at week 52 (Post-TB) and healthy volunteers (non-TB)), adjusted for sex, age and height, versus the South African standard for the calculation of z-scores [23]

We compared the lung function of the TB patients at week 52 with that of 155 healthy volunteers. The main characteristics of the TB cohort versus healthy volunteers are summarized in S3 table. There was a significant lower proportion of men (40.65% versus 67.7%), smokers (12.3% versus 37.1%) and HIV-positives (26.5%, self-reported, versus 62.9%) among the healthy volunteers, *p* < 0.001. In Fig. 4, the density distribution of adjusted z-scores for FVC and FEV1 are shown for both study groups together with the South African reference standard. Compared to the healthy volunteers, the TB cohort demonstrated a much more pronounced shift of both curves (FVC and FEV1) to lower z-scores and also a large left-side tail below a z-score of − 1.64, which represents a higher proportion of subjects with abnormal lung function.

## Discussion

In this prospective study, the majority of pulmonary TB patients had abnormal lung function in spirometry during and after TB treatment. This finding is in line with several other publications and our own meta-analysis (unpublished data) showing that up to 70% of previous TB patients suffer from chronic and clinically relevant lung function impairment [3, 5, 11, 32]. Currently, there is not much data available on the long term medical but also socio-economic consequences of chronic lung damage after TB [19]. It is very likely, that many patients with LI presenting with continuous symptoms might be incorrectly classified as recurrent TB cases and referred to unnecessary TB treatment. In the majority, however, LI remains undiagnosed and untreated, possibly resulting in increased mortality compared to the standard population [33, 34].

Due to the prospective study design, lung function could be measured during and after TB treatment. Like in few other prospective studies [12, 35], an increase in FVC and FEV1 over time was observed in most subjects that in young adults might be related to muscular and cardiovascular improvement after TB cure. However, this improvement was usually not significant and led to a normalization of lung function in only 15% of subjects with LI at week 8. In general, almost all participants with an abnormal lung function 1 year after TB treatment, already had lung function impairment early during treatment. The data indicate that potential medical interventions aiming to preserve lung function should probably be applied rather early during TB treatment. Currently, there are no guidelines on the diagnosis, follow up and treatment of patients with post TB lung disease [8]. In order to develop concrete therapeutic strategies, we need a better understanding of the heterogeneity of pulmonary disease after TB and the underlying patho-mechanisms [19, 36]. Most likely, future preventive treatment options will depend on the causal pathway and/or the prevailing type of LI [36].

It is worth noting that opposed to the findings in other studies from Africa [5, 11, 18, 32], almost all our participants had pulmonary restriction (low FVC) after TB and not pulmonary obstruction (low FEV1/FVC-ratio). Ravimohan et al. described different, genetically defined, immunological pathways that are defining lung outcome, including the type of ventilation impairment [37]. In addition to the genetic constitution of our study population, also the composition of other background risks such as (indoor) air pollution, prevalence of childhood pulmonary infections or risk behaviour could be different to other study populations and thus, could explain the high prevalence of pulmonary restriction. Further, age per se is a risk factor for chronic lung diseases and especially Chronic Obstructive Respiratory Disease (COPD). With a median age of about 30 years, our study cohort was relatively young. Therefore, the prevalence of obstruction could also be lower than in other studies.

Despite the small sample size, we performed univariable regression analysis in order to get an insight into possible risk factors for LI after TB. The results indicate that factors that might be related to a stronger inflammatory response in study participants (BMI < 18.5, CD4 ≥ 200/μl, low haemoglobin) increase the risk for LI. Further, cigarette smoking and alcohol drinking beyond a certain threshold (e.g., ≥10PY, 150 g/80 g alcohol per week) were also increasing the risk for long term LI. Possible causal pathway could either be via direct damage of lung tissue or immune cells or due to poorer health seeking behaviour. Also, a certain level of pre-existing lung damage cannot be excluded in heavy smokers and alcohol drinkers (see below). The finding that light smokers have a lower risk for LI could be explained by the fact that those who can afford to buy cigarettes but do not smoke much may have better access to health care or better physical conditions (related to wealth) that are protective of LI after TB. Reasons for the association of female sex with LI could be differences in immune response, nutrition status, delayed access to medical care, higher exposure to indoor air pollution, etc., compared to men. We did not find any association between HIV-status and LI in our study. Previous research found contradicting evidence for sex, smoking behaviour and also HIV-status as risk factors for TB lung outcome [3, 5, 10–18]. With regards to the role of HIV-coinfection for the development of LI, one reason for the heterogeneity of findings across the few available studies could be the differences in the main type of LI found. While it could be shown that HIV may be associated with obstructive lung disease [14, 38, 39], its role in the development of restrictive lung impairment, which was the dominant LI type of our study, is not known. Interestingly, an association of increasing CD4 counts with reduced FEV1 was also observed in another study [37]. However, like this manuscript, the majority of existing publications was based on rather small study cohorts which usually prevented sub-group analyses or assessment of bias such as confounding and effect modification.

In the context of data analysis, we faced several challenges: in the absence of a Mozambican spirometric reference standard, we used South African prediction equations as basis for the analysis of spirometry data (except for FEV1/FVC ratio) as there was an even poorer fit with the reference values for Africans published by the Global Lung Initiative (unpublished data). In order to get an indication to what extent the South African standard is applicable to our TB study population in Maputo we collected lung function data in healthy volunteers (no TB in the past, no chronic or acute lung disease) from the same neighbourhood as the TB patients. The slight shift to lower lung volumes in the healthy volunteers compared to the South African standard (Fig. 4) could either mean that there is a certain level of pre-existing LI in the study population or that the South African standard is not ideal for the population in Maputo. In any case, the data suggest that the observed LI in previous TB patients is largely related to TB, especially in those patients with relatively low z-scores, representing moderate or severe abnormalities in spirometry. In this article, we present the results for FVC in addition to FEV1 in order to avoid the impression that our study participants suffer from airway obstruction. In the majority of them, however, FEV1 was reduced as a consequence of low FVC. In the absence of clinical guidelines on post TB LI, it is unclear whether spirometry (alone) is suitable to identify those patients who suffer most from post TB lung disease or would benefit most from pulmonary follow up and treatment. It might be that additional investigations, e.g., chest x-ray, cardiac investigation, exercise tests or symptoms screens, are needed to identify patients with manifest comorbidities of LI in order to prioritize supportive treatment, given that the underlying pulmonary damage is currently not curable.

A recent publication showed that reduced, but still normal, values for FEV1 of above -2SD of predicted were associated with significantly increased mortality [9]. This supports the strategy to use spirometry to identify patients with LI after TB who are possibly at highest risks for increased morbidity and mortality. However, there is a need of studies in larger cohorts to firstly get more robust data on the prevalence, type and severity of LI in previous TB patients. Secondly, the role of risk factors and the underlying causal pathways need to be addressed in those studies in order to develop strategies for treatment and prevention of LI. Finally, as the basis for future research, guidelines on measurement and classification of LI as well as agreed strategies on data analysis are urgently needed.

## Conclusions

Moderate or severe LI was observed in a third of cured TB patients in this study. The majority of patient presented with pulmonary restriction (low FVC). Female sex, low haemoglobin and heavy smoking were associated with LI in our study population. Further research is urgently needed to gain deeper insight into the development and characteristics of post TB LI, the causal pathways and potential treatment strategies.

## Supplementary information

**Additional file 1 S1 table.** Inclusion and exclusion criteria of healthy volunteers. **S2 table.** Distribution of risk factors in participants from TB cohort without and with LI at week 52 (and with mild or moderate/severe LI among those with LI). **S3 table.** Baseline characteristics of TB cohort and healthy volunteers. **S1 fig.** z-scores for FVC (1a) and FEV1 (1b) at week 8, 26 and 52 of those participants with normal values for FVC and FEV1 at week 8.

## Data Availability

The datasets used and/or analysed during the current study are available from the corresponding author on reasonable request.

## Abbreviations

ATS: American Thoracic Society
BMI: Body Mass Index
COPD: Chronic Obstructive Respiratory Disease
CNBS: Comité Nacional de Bioética para Saúde
ERS: European Respiratory Society
FEV1: Forced Expiratory Volume in 1 s
FVC: Forced Ventilatory Capacity
GLI: Global Lung Initiative
HIV: Human Immunodeficiency Virus
INS: Instituto Nacional de Saúde
IQR: Interquartile range
LLN: Lower limit of normality
LI: Lung Impairment
Mtb: *Mycobacterium tuberculosis*
NTP: National TB Program
RRR: Relative Risk Ratios
SD: Standard Deviation
TB: Tuberculosis
