# Supplementary material for: Development of chronic lung impairment in Mozambican TB patients and associated risks
Source: BMC Pulm Med. 2020 May 7;20:127. doi: 10.1186/s12890-020-1167-1 (PMC7203866; doi:10.1186/s12890-020-1167-1)
Supplement: Supplementary file 1 — Additional file 1 S1 table. Inclusion and exclusion criteria of healthy volunteers. S2 table. Distribution of risk factors in participants from TB cohort without and with LI at week 52 (and with mild or moderate/severe LI among those with LI). S3 table. Baseline characteristics of TB cohort and healthy volunteers. S1 fig. z-scores for FVC (1a) and FEV1 (1b) at week 8, 26 and 52 of those participants with normal values for FVC and FEV1 at week 8. [file 12890_2020_1167_MOESM1_ESM.docx]

**S1 table. Inclusion and exclusion criteria of healthy volunteers**

| **Inclusion criteria** | **Exclusion criteria** |
| --- | --- |
| ≥ 18 years old | TB suspect and/or TB symptoms present (at least 2 symptoms or signs) * |
| Able and willing to give informed consent to participate in spirometric investigation | Past TB disease or anti-TB treatment |
|  | Any contraindication for spirometry** |
|  | Any current or past (chronic) respiratory disease*** |

Legend to supplementary table 1:

*TB symptoms: productive cough > 2 weeks, fever > 2 weeks, night sweats > 2 weeks, lasting weight loss

**Contraindication for spirometry: pneumothorax, eye or brain surgery (last 8 weeks), major surgery (last 6 weeks), heart attack, lung embolism or angina pectoris (last 4 weeks), respiratory infection (e.g. chest cold, pneumonia, bronchitis in last 3 weeks), smoked tobacco (last hour), heavy meal (last hour), albuterol rescue inhaler (Salbutamol, Salmeterol in last hour), wearing any tight clothing that interferes with ability to breathe deeply, wearing dentures.

***Respiratory diseases: Asthma or COPD diagnosed by medical personnel, general wheezing or whistling in chest (last 2 years), respiratory infection (e.g. chest cold, pneumonia, bronchitis in last 3 weeks), breathing difficulties during mild exercise, or at night.

**S2 table. Distribution of risk factors in participants from TB cohort without and with LI at week 52 (and with mild or moderate/severe LI among those with LI).**

| **Risk factor, total N= 62** | **Total** | **No LI,**  **% (n)**  **(N=22)** | **Any LI,**  **% (n)**  **(N=40)** | **Mild LI,**  **(n)**  **(N=18)** | **Moderate/**  **severe LI,**  **(n)**  **(N=22)** |
| --- | --- | --- | --- | --- | --- |
| **Sex** |  |  |  |  |  |
| Male | 42 | 45.24 (19) | 54.76(23) | 10 | 19 |
| Female | 20 | 15.00 (3) | 85.00 (17) | 8 | 9 |
| **Age** |  |  |  |  |  |
| <40 years old | 46 | 34.78 (16) | 65.22 (30) | 15 | 15 |
| ≥40 years old | 16 | 37.50 (6) | 62.50 (10) | 3 | 7 |
| **BMI** |  |  |  |  |  |
| <18.5 | 26 | 30.77 (8) | 69.23 (18) | 10 | 8 |
| ≥18.5 | 36 | 38.89 (14) | 61.11 (22) | 8 | 14 |
| **HIV-status** |  |  |  |  |  |
| Positive | 39 | 33.33 (13) | 66.67 (26) | 13 | 13 |
| CD4- cells < 200/µl | 18 | 44.44 (8) | 55.56 (10) | 4 | 6 |
| CD4- cells ≥ 200/µl | 21 | 23.81 (5) | 76.19 (16) | 9 | 7 |
| Negative | 23 | 39.13 (9) | 60.87 (14) | 5 | 9 |
| **Culture Conversion at week 8** |  |  |  |  |  |
| Yes | 35 | 42.86 (15) | 57.14 (20) | 10 | 10 |
| No | 27 | 25.93 (7) | 74.07 (20) | 8 | 12 |
| **Culture conversion at week 26** |  |  |  |  |  |
| Yes | 58 | 36.21 (21) | 63.79 (37) | 16 | 21 |
| No | 4 | 25 (1) | 75.00 (3) | 2 | 1 |
| **Ever smoked** |  |  |  |  |  |
| Yes | 22 | 54.54 (12) | 45.46 (10) | 2 | 8 |
| No | 40 | 25.00 (10) | 75.00 (30) | 16 | 14 |
| **Pack Years***^,^ ** |  |  |  |  |  |
| <10 | 13 | 69.23 (9) | 30.77 (4) | 1 | 3 |
| ≥10 | 7 | 28.57 (2) | 71.43 (5) | 0 | 5 |
| **Critical alcohol consumption*^,^ **** |  |  |  |  |  |
| Yes | 32 | 40.63 (13) | 59.38 (19) | 6 | 13 |
| No | 30 | 30.00 (9) | 70.00 (21) | 12 | 9 |
| **C-reactive protein*** |  |  |  |  |  |
| <100 mg/dl | 41 | 31.71 (13) | 68.29 (28) | 12 | 16 |
| ≥100 mg/dl | 17 | 41.18 (7) | 58.82 (10) | 5 | 5 |
| **Haemoglobin** |  |  |  |  |  |
| Median male, g/dl (IQR) | 42 | 11.80 (11.05,12.65) | 11.20  (9.80, 12.60) | 11.65 (9.38,13.05) | 11.2 (10.20,12.10) |
| Median female, g/dl (IQR) | 20 | 11.3  (11.10, 12.70) | 9.80 (9.40,10.70) | 9.65  (8.10, 10.18) | 9.90  (9.50, 10.70) |
| **Overall affected lung at baseline, Ralph et al.**** |  |  |  |  |  |
| Median, % (IQR) | 62 | 17.5  (15,20) | 15  (14,22.50) | 15 (10,23.75) | 20  (15, 20) |

Legend to supplementary table 2:

*missing observations: CRP for 4 participants, Pack Years (PY) for 2 participants, amount of alcohol consumption for 2 participants.

**definitions:

- Anaemia, definition according to WHO, non-anaemia: 12mg/dl or higher (women) or 13 mg/dl or higher (men), mild: 11.0-11.9 g/dl (women) and 11.0-12.9 g/dl (men), moderate: 8.0g/dl-10.9g/dl (both sexes), severe: <8.0g/dl (both sexes)[1].
- X-ray scoring system for affected lung, according to Ralph et al [2].
- Pack Years: numbers of years a person smoked 1 pack (20 cigarettes) per day, ≥10 pack years means that the respective study participant smoked an equivalent of one pack of cigarettes every day for 10 years (e.g. 1 pack of cigarettes for 10 years, or 2 packs of cigarettes for 5 years, or ½ packs of cigarettes for 20 years) at baseline study visit.
- Critical alcohol consumption: in men (women) more than 60g (30g) alcohol per occasion of alcohol drinking and/or more than 150g (80g) alcohol per week, according to the International Alliance for Responsible Drinking for general population [3].

**S3 table. Baseline characteristics of TB cohort and healthy volunteers**

| **Characteristic** | **TB cohort**  **(N=62)** | **Healthy volunteers**  **(N=155)** | **p-value**** |
| --- | --- | --- | --- |
| **Sex** |  |  |  |
| Male, % (n/N) | 67.74 (42/62) | 40.65 (63/155) | <0.001 |
| **Age,** median (IQR) | 29.5 (25, 40) | 33.9 (26, 42) | 0.13*** |
| < 40 years old, % (n/N) | 74.19 (46/62) | 67.74 (105/155) | 0.35 |
| **HIV-status*** |  |  |  |
| Positive, % (n/N) | 62.90 (39/62) | 26.45 (41/155) | <0.001 |
| **History of TB** |  |  |  |
| Yes, % (n/N) | 6.45 (4/62) | 0.00 (0/155) | 0.001 |
| **Ever smoked** |  |  |  |
| Yes, % (n/N) | 37.10 (23/62) | 12.26 (19/155) | <0.001 |

Legend to supplementary table 3:

*missing/unknown self-reported HIV-status in 47 (47 of 155, 30.32%) subjects of healthy volunteers, **p-value for difference in proportions from chi-square test, *** rank sum test.

**S1 fig. z-scores for FVC (1a) and FEV1 (1b) at week 8, 26 and 52 of those participants with normal values for FVC and FEV1 at week 8**


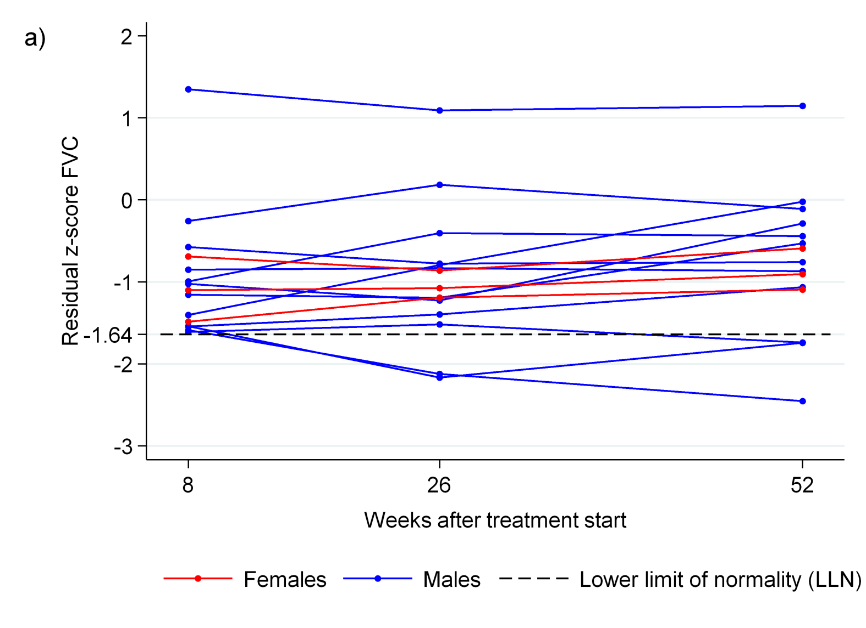

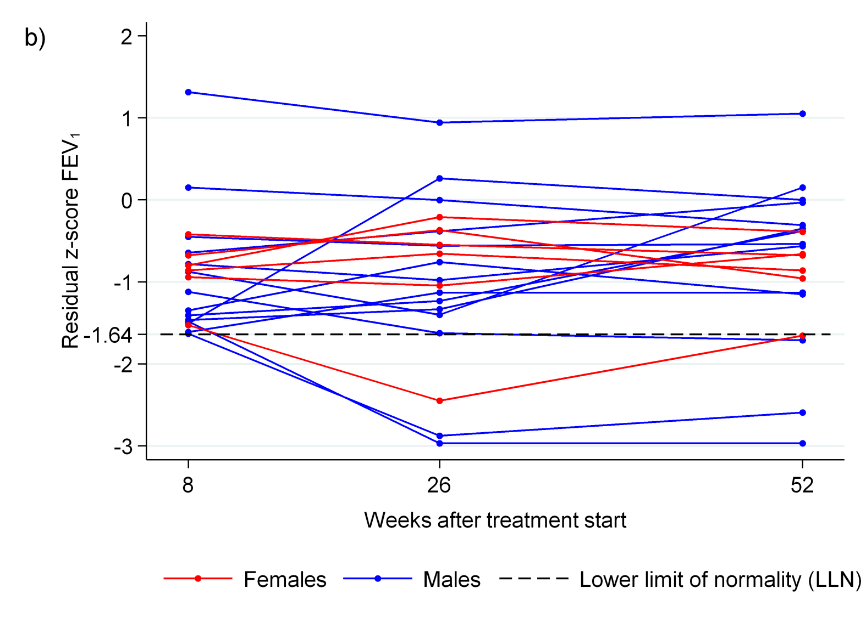


**Legend to supplementary figure 1:** Change in residual z-scores for FVC (1a) and FEV1 (1b) over time in study participants without LI (residual z-score > 1.64) at week 8. Residual z-scores (adjusted for sex, height and age) were calculated based on South African standard values (and prediction equations).

1. Organization WH. Haemoglobin concentrations for the diagnosis of anaemia and assessment of severity. World Health Organization, 2011.

2. Ralph AP, Ardian M, Wiguna A, Maguire GP, Becker NG, Drogumuller G, Wilks MJ, Waramori G, Tjitra E, Sandjaja, Kenagalem E, Pontororing GJ, Anstey NM, Kelly PM. A simple, valid, numerical score for grading chest x-ray severity in adult smear-positive pulmonary tuberculosis. *Thorax* 2010: 65(10): 863-869.

3. (IARD) IAfRD. Drinking guidelines: General population. 2018 [cited 2018 June]; Available from: ://[www.iard.org/resources/drinking-guidelines-general-population/](file:///C:\Users\Celso%20Khosa\Desktop\2.PhD_LMU_2014-2017\RP%20IV%202018\Manuscript\Thorax\Final\www.iard.org\resources\drinking-guidelines-general-population\)
